# Prenatal Evaluation of Genetic Abnormalities in Fetuses With Single Umbilical Artery: A Retrospective Cohort Study

**DOI:** 10.1155/genr/3827416

**Published:** 2026-08-23

**Authors:** Yuqin Chen, Xiaoqing Wu, Meiying Cai, Linjuan Su, Xiaorui Xie, Hailong Huang, Rongzhao Zhang, Liangpu Xu

**Affiliations:** ^1^ Medical Genetic Diagnosis and Therapy Center, Fujian Maternity and Child Health Hospital College of Clinical Medicine for Obstetrics & Gynecology and Pediatrics, Fujian Medical University, Fuzhou 350001, China, fjmu.edu.cn; ^2^ Center for Experimental Research in Clinical Medicine, Fujian Provincial Hospital, Fuzhou 350001, China, fjsl.com.cn

**Keywords:** chromosomal abnormalities, fetal malformations, prenatal diagnosis, single umbilical artery

## Abstract

**Objective:**

To investigate the genetic factors associated with fetal single umbilical artery (SUA) and concomitant structural anomalies.

**Methods:**

A retrospective review was performed on the ultrasound characteristics of 375 SUA fetuses diagnosed by color Doppler ultrasound at Fujian Provincial Maternity and Children’s Hospital from February 2010 to February 2022. Single‐nucleotide polymorphism (SNP) array and karyotype analysis were performed to evaluate the relationship between chromosomal abnormalities and isolated/nonisolated SUA.

**Results:**

Most pregnant women were aged 25–35 years, and 210 were primigravida or nulliparous women with a history of miscarriage. Among 375 fetuses, 101 had isolated SUA (iSUA), 116 had SUA with additional abnormal ultrasound soft markers, and 158 had SUA with congenital structural malformations (predominantly cardiovascular and genitourinary). The chromosomal karyotype abnormality rate was 16.53%, significantly higher in the SUA with multiple system malformation group. SNP‐array testing in 152 cases identified 11 cases of chromosome number abnormalities and 13 microdeletions or microduplications. Of the 131 cases with successful follow‐up, 77 term live births, 49 elective terminations, 2 preterm births, and 3 deaths. All 29 cases in the SUA with multiple system malformation group were terminated.

**Conclusions:**

SUA was most common in pregnant women aged 25–35 and significantly higher in primigravidas and nulliparous women with a history of miscarriage. Female fetuses had a higher SUA incidence than males. Chromosomal abnormality rates increased with associated malformation number and occurred even in iSUA. SNP‐array offers a higher resolution in detecting chromosomal microdeletions or microduplication syndromes compared to conventional karyotyping.

## 1. Introduction

The umbilical cord plays a vital role in connecting the fetus to the placenta. Structurally, the umbilical cord normally comprises three blood vessels: a single vein that transports nutrients and oxygen from the placenta to the fetus, and two arteries that carry fetal metabolic waste to the placenta. However, anatomical abnormalities can result in the presence of a single artery and vein within the umbilical cord; this condition is referred to as a single umbilical artery (SUA). Typically, it is the most common anatomical anomaly of the umbilical cord. This condition often results from several causes, including occlusion or secondary atrophy of one umbilical artery, occurrence of primary hypoplasia of the umbilical artery, or a single persistent urachal artery [1]. The incidence of SUA is approximately 0.5–5%, with associated risk factors, including advanced maternal age, smoking, presence of metabolic disorders, assisted reproduction, and multiple pregnancies [2–4].

Notably, SUA is a prenatal ultrasound soft marker associated with elevated risk for fetal congenital malformations, chromosomal abnormalities, preterm birth, and intrauterine growth restriction. Typically, SUA is accompanied by hemodynamic changes, resulting in irregular blood flow, which affects the supply of nutrients and oxygen to the fetus. Insufficient supply of nutrients and oxygen to the fetus is associated with the occurrence of developmental abnormalities across multiple fetal systems, particularly the cardiovascular, genitourinary, and digestive systems. These abnormalities elevate the risk of adverse outcomes, including malformations, preterm birth, and intrauterine growth restriction. This condition is referred to as nonisolated SUA (niSUA) [5]. Conversely, isolated SUA (iSUA) is defined as the absence of additional abnormalities on prenatal ultrasound. Additionally, SUA is not only closely associated with fetal structural abnormalities but also with chromosomal abnormalities, including both trisomy 13 and 18 [2, 6]. Notably, SUA and the presence of multiple structural abnormalities are positively correlated with the occurrence of chromosomal abnormalities, in comparison to iSUA cases [4].

Although SUA is a known risk factor for chromosomal abnormalities, most previous studies have been limited by the use of conventional karyotyping alone, which fails to detect submicroscopic copy number variations. To address this knowledge gap, this study aims to investigate the correlation between fetal SUA, concomitant structural abnormalities, and chromosomal abnormalities using both comprehensive karyotype analysis and SNP‐array technology. Furthermore, the study aims to investigate the impact of these factors on pregnancy outcomes. These findings will provide a theoretical foundation that informs genetic counseling and effective prenatal diagnosis to improve pregnancy outcomes and promote healthy child delivery.

## 2. Materials and Methods

### 2.1. Study Subjects

This was a retrospective study that enrolled a total of 375 fetuses diagnosed with SUA using color Doppler ultrasound at Fujian Provincial Maternity and Children’s Hospital from February 2010 to February 2022 by reviewing the hospital’s ultrasound database and medical records. Informed consent was obtained from all pregnant participants at the Medical Genetics Diagnostic and Treatment Center before administration of amniocentesis or cordocentesis. General clinical data such as the age of pregnant women undergoing invasive prenatal diagnosis, gestational age, and obstetric history were collected from medical records. Karyotype analysis was conducted for all the cases. However, due to the absence of single‐nucleotide polymorphism (SNP) array testing at our center before June 2016, this technique was performed in only 152 fetuses. Pregnant women diagnosed with conditions such as gestational diabetes, autoimmune diseases, preeclampsia, uterine artery Doppler abnormalities, and other related diseases were excluded from this study. Follow‐up information, including mode of delivery, neonatal gender, birth weight, and subsequent growth and development, was obtained retrospectively through telephone interviews and review of hospitalization records. This study was approved by the Ethics Committee of Fujian Provincial Maternity and Children’s Hospital, with all experimental procedures conducted in strict adherence to the relevant guidelines and regulations.

### 2.2. SUA Diagnostic Criteria

Multiple ultrasound examinations are routinely performed throughout pregnancy, including a comprehensive fetal anatomical survey before 24 weeks for structural anomaly screening, and follow‐up scans after 24 weeks for late‐onset malformations and fetal growth. Diagnosis was conducted using color Doppler ultrasound equipment equipped with a 4–8 MHz abdominal probe. Following successful diagnosis of SUA, a certified prenatal ultrasound specialist conducted a comprehensive ultrasound examination, which included sequential assessment of the fetal brain, face, neck, chest, abdomen, spine, limbs, placenta, and amniotic fluid. In cases where structural abnormalities were observed, the diagnosis was jointly reviewed by a senior associate chief physician or a more experienced specialist. The final diagnosis of fetal malformations was based on findings from autopsy or postnatal clinical examination. In cases where neither was available, prenatal ultrasonographic results were used as the diagnostic reference.

### 2.3. SUA Grouping

The fetuses were classified based on coexisting ultrasound findings into three groups: isolated SUA, SUA with additional soft markers, and SUA with malformations. Isolated SUA is considered a diagnosis of exclusion, characterized by the absence of additional abnormalities on prenatal ultrasound examination. Soft markers associated with SUA include increased nuchal translucency or thickening of the neck skin folds, nasal bone hypoplasia, choroid plexus cysts, echogenic foci in the heart, widened posterior fossa, enhanced intestinal echoes, mild renal pelvis dilation, short femur, and short humerus. Fetal malformations associated with SUA include defects of the central nervous system, such as cerebellar hypoplasia, anencephaly, corpus callosum hypoplasia, and meningocele; defects of the genitourinary system, including renal hypoplasia, multicystic dysplastic kidneys, ectopic kidneys, polycystic kidneys, and megacystis; cleft lip and palate; gastrointestinal defects, such as esophageal atresia or stenosis, duodenal obstruction, and small bowel obstruction; defects of the abdominal wall, including gastroschisis and omphalocele; thoracic defects, such as diaphragmatic hernia and pulmonary hypoplasia; cardiac and great vessel defects, including tetralogy of Fallot, ventricular septal defects, hypoplastic left heart syndrome, persistent left superior vena cava, right aortic arch, and double outlet right ventricle; and spinal and limb defects, including spina bifida, scoliosis, and polydactyly.

### 2.4. Karyotype Analysis

Amniotic fluid and umbilical cord blood samples were cultured and processed for chromosomal analysis following the standard G‐banding protocol employed in our laboratory. A minimum of 20 metaphase cells were examined, with detailed karyotypic analysis performed on at least five metaphases. In cases where mosaic abnormalities were suspected, additional metaphase spreads were analyzed to confirm the findings. The karyotypes were interpreted at a resolution of 320–500 bands per haploid set and analyzed according to the 2020 International System for Human Cytogenetic Nomenclature (ISCN2020).

### 2.5. DNA Extraction

Genomic DNA was extracted from amniotic fluid and umbilical cord blood samples using the QIAGEN genomic DNA extraction kit (QIAGEN, Germany) and preserved at −20°C for subsequent analysis.

### 2.6. SNP‐Array

The Affymetrix CytoScan 750K Array (Affymetrix Inc., Santa Clara, CA, USA) was used to conduct the SNP genotyping. Notably, DNA digestion, amplification, purification, fragmentation, labeling, hybridization, staining, and scanning were performed according to the manufacturer’s instructions. Data were then analyzed using the Chromosome Analysis Suite (ChAS) software, with the genomic findings interpreted relative to the GRCh37 (Human Genome 19, HG19) human genome reference assembly. All detected copy number variations (CNVs) were compared with both the internal and public national CNV databases, including the Database of Genomic Variants (DGV), the DECIPHER database (Human Chromosome Imbalance and Phenotype Database using combined resources), the International Standard for Cytogenomic Arrays (ISCA), and Online Mendelian Inheritance in Man (OMIM). Based on the guidelines provided by the American College of Medical Genetics and Genomics (ACMG), the SNP‐array results were classified into five levels: pathogenic, likely pathogenic, variants of uncertain significance (VUS), likely benign, and benign [7]. Notably, both pathogenic and likely pathogenic variants were considered to be clinically significant findings. Parental SNP‐array was recommended to determine the genetic inheritance of CNVs.

### 2.7. Follow‐Up

Hospitalization records and telephone interviews were reviewed to conduct follow‐up analysis. Information recorded during the follow‐ups included the mode of delivery, neonatal gender, birth weight, and subsequent growth and development. The final follow‐up was in August 2023.

### 2.8. Statistical Analysis

All data processing and analyses were conducted using SPSS version 23.0, with graphical representations generated using GraphPad Prism 7.0. Categorical data were presented as frequencies or percentages, with group comparisons performed using the chi‐squared test. Continuous data were expressed as mean ± standard deviation, with comparisons between two groups conducted using independent sample *t*‐tests. Analysis of variance (ANOVA) was used for comparison involving three or more groups. A *p*‐value of < 0.05 was considered statistically significant.

## 3. Results

### 3.1. Comparison of General Clinical Data

A total of 375 cases of fetal SUA were diagnosed via prenatal ultrasonographic imaging from February 2010 to February 2022. Notably, 168 were male fetuses (44.8%) and 207 were female fetuses (55.2%). Additionally, the average maternal age was 29.25 ± 4.99 years, with a range of 18–42 years; majority of the cases, 230, occurred in participants within the range of 25–35 years; 94 cases were under 25 years; and 51 cases over 35 years (*p* < 0.001), with these differences being statistically significant. Ultrasound examination results revealed an average fetal age of 24.47 ± 3.78 weeks, with a range of 16–40 weeks; specifically, 208 fetuses were 24 weeks or below, while 167 were older than 24 weeks (*p* < 0.001), with these differences being statistically significant. A total of 165 women were parous, while the remaining 210 were nulliparous (including primigravida and those with a history of miscarriage only) (*p* < 0.001) (Table 1), with the statistical findings indicating significant differences between the two groups. The results indicate a higher prevalence of SUA among female fetuses, with the highest occurrence observed in pregnancies of women aged 25–35 years. Increased detection rate was observed in cases with a gestational age of ≤ 24 weeks and among primigravida or women with a history of miscarriage.

**TABLE 1:** Comparison of perinatal data.

Group	*N*	*p*
Age		
≤ 25	94	< 0.001
25–35	230	
> 35	51	
Gestational age		
≤ 24	208	< 0.001
> 24	167	
Parity		
0	165	< 0.001
≥ 1	210	
Fetuses		
Male	168	—
Female	207	

*Note:* Independent sample *t*‐tests were used for comparison between two groups; ANOVA was used for comparison involving three or more groups. A *p*‐value of < 0.05 was considered statistically significant.

### 3.2. SUA With Associated Structural Abnormalities

Among the 375 cases, 101 fetuses exhibited iSUA, while 274 fetuses exhibited SUA concomitant with other structural abnormalities. Among the latter group, 116 fetuses exhibited the occurrence of additional ultrasound soft markers, while 158 fetuses had other structural malformations. Additionally, among the fetuses with malformations, 94 had malformation in a single system, while 64 exhibited malformations in two or more systems. The most prevalent malformations included cardiovascular defects occurring in 102 cases, genitourinary defects in 39 cases, skeletal defects in 29 cases, cranial malformations in 26 cases, gastrointestinal defects in 25 cases, facial malformations in 8 cases, and other defects (including omphalocele, umbilical hernia, and cervical cystic hygroma) occurring in 18 cases (Table 2).

**TABLE 2:** SUA with associated structural abnormalities.

Group	*N*
Cardiovascular system malformations	102
Genitourinary system malformations	39
Skeletal malformations	29
Cranial malformations	26
Gastrointestinal malformations	25
Facial malformations	8
Others	18

### 3.3. Chromosomal Abnormalities in Fetuses With SUA

Among the 375 fetuses diagnosed with SUA who underwent karyotypic analysis of the amniotic fluid or cord blood samples, 62 cases exhibited chromosomal abnormalities, resulting in a karyotype abnormality rate of 16.53%. Notably, a significantly higher chromosomal abnormality rate was observed in the group with associated malformations compared to that of the iSUA group (*p* < 0.001). Additionally, the multiple system malformation group exhibited a significantly higher rate of abnormalities compared to the other groups (Table 3). Among the 152 cases that underwent SNP‐array testing, 24 exhibited the presence of abnormalities: 7 cases of trisomy 18, 2 cases of trisomy 21, 1 case of trisomy 13, 1 case of 45, X, and 13 cases of microdeletions or microduplications (Table 4). The SNP abnormality rate exhibited the absence of significant differences between the groups, an observation that is potentially due to the limited sample size. However, the multiple system malformation group exhibited a significantly higher abnormality rate compared to the other groups (Table 5).

**TABLE 3:** Karyotype abnormalities in fetuses with SUA.

Group	*N*	Normal	Abnormal	*χ* ^2^, *p*
iSUA	101	93	8	
SUA with ultrasound soft markers	116	102	14	1.020, 0.313
SUA with one system malformation	94	78	16	3.736, 0.053
SUA with multiple system malformations	64	40	24	21.926, < 0.001

*Note:* Chi‐squared test. A *p*‐value of < 0.05 was considered statistically significant.

**TABLE 4:** SNP‐array results of 13 SUA cases.

Case	Prenatal sample	GA	SNP array	Size	Gene symbols	Classification of variants	Karyotype	Pregnancy outcome
1	Amniotic Fluid	26	arr [hg19] 5q14.2 (81,733,061–82,762,577) x1	1.0 Mb	SCARNA18, XRCC4	VUS	46,XY	Live birth

2	Amniotic Fluid	25	arr [hg19] 15q25.2q25.3 (85,053,133–85,711,127) x3	658 Kb	SCAND2P, WDR73, etc.	VUS	46,XY	Live birth

3	Amniotic Fluid	18	arr [hg19] 5q23.15q23.2 (120140200–121429808) x1	1.29 Mb	FTMT, SRFBP1, etc.	VUS	46,XY	—

4	Amniotic Fluid	18	arr [hg19] Xp22.33p11.21 (168,551–56,661,860) x1; Xq21.1q28 (79,764,187–155,233,098) x1	56 Mb; 75 Mb	STS, KAL1, etc; ATRX, POU3F4, etc	P; P	46,X,+mar [21]/45,X [12]	TOP

5	Amniotic Fluid	21	arr [hg19] 1q21.1q21.2 (146096700–147391923) x3	1.3 Mb	GJA5, GJA8, etc	P	46,XY	—

6	Amniotic Fluid	19	arr [hg19] 22q11.1q11.21 (16,888,899–18,649,190) x4	1.7 Mb	CECR1, CECR2, etc	P	47,XY,+mar	TOP

7	Amniotic Fluid	24	arr [hg19] 17p11.2 (18,722,449–20,225,861) x3	1.5 Mb	GRAP, B9D1, etc	LB	46,XX	Live birth

8	Amniotic Fluid	24	arr [hg19] 16q22.2q23.2 (7146398–79614082) x3	8.15 Mb	DHODH, CHST6, etc	VUS	46,XY,inv (9) (p12q13)	—

9	Amniotic Fluid	21	arr [hg19] Xp22.33p11.22 (168,551–52,819,260) x1	52.6 Mb	SHOX, MAOA, etc	P	46,X, psu idic (X) (p11.22) [40] /45,X [10]	TOP

10	Amniotic Fluid	22	arr [hg19] 15q13.2q13.3 (31,104,220–32,444,043) x1	1.3 Mb	FAN1, TRPM1, etc	LP	46,XY	TOP

11	Amniotic Fluid	24	arr [hg19] 4p16.3p15.31 (68,345–20,522,754) x1	20.4Mb	WHSC1, WHSC2, etc	P	46,XY,del (4) (p15.3)	TOP

12	Amniotic Fluid	18	arr [hg19] 17p11.2 (20,641,987–21,508,918) x3; 17q24.3q25.1 (70,731,144–72,248,511) x4; 17q25.1q25.3 (72,248,511–75,448,070) x3	867 Kb; 1.5 Mb; 3.2 Mb	USP22, DHRS7B, etc; KCNJ16, KCNJ2, etc; COG1, SEPTIN9, etc	VUS; LP; LP	47,XX,+mar [16] /46,XX [18]	TOP

13	Cord Blood	28	arr [hg19] 8q24.13 (126,044,027–126,414,021) x3	370 Kb	NSMCE2, TRIB1, etc	—	46,XY	—

*Note:* P, pathogenic.

Abbreviations: GA, gestational age; LB, likely benign; LP, likely pathogenic; TOP, termination of pregnancy; VUS, variants of uncertain significance.

**TABLE 5:** SNP‐array abnormalities in fetuses with SUA.

Group	*N*	Normal	Abnormal	*χ* ^2^, *p*
iSUA	32	28	4	
SUA with ultrasound soft markers	55	48	7	0.001, 0.975
SUA with one system malformation	42	36	6	0.050, 0.824
SUA with multiple system malformations	24	17	7	2.413, 0.120

*Note:* Chi‐squared test. A *p*‐value of < 0.05 was considered statistically significant.

### 3.4. Pregnancy Outcomes in Pregnant Women With SUA

Among the 375 fetuses diagnosed with SUA, 131 cases were successfully followed up. Among them, 77 resulted in normal births and 49 were electively terminated, 2 cases were preterm, and 3 cases were neonatal deaths. In the iSUA group, 4 pregnancies were terminated. Karyotype analysis revealed that 2 had trisomy 18, 1 had a karyotype of 46,XY,inv(9) (p12q13) (a common pericentric inversion generally considered a benign variant), and 1 had a normal karyotype. The termination in the normal karyotype case was due to subsequent ultrasound findings of fetal structural anomalies, despite the normal karyotype result. In the group of SUA with other ultrasound soft markers, 5 cases were electively terminated, with karyotype analysis revealing trisomy 18 in 2 cases, +mar mosaicism in 2 cases, and 47, XXX/45, X mosaicism in 1 case. Mortality occurred in three cases, with all three exhibiting normal chromosomal results, and follow‐up analysis indicated that the deaths occurred several days postpartum, with one suspected of succumbed to rubella. In the group with one system malformation, 11 cases were electively terminated, with karyotype analysis revealing trisomy 18 in 3 cases, +mar mosaicism in 1 case, 47,XY,+8/46, XY mosaicism in 1 case, and normal karyotypes in 6 cases. In the group with multiple system malformations, all 29 cases were electively terminated, with karyotype analysis exhibiting trisomy 18 in 7 cases (including 2 exhibiting inversion of chromosome 9), trisomy 13 in 2 cases, Y inversion in 1 case, 46, X, psu idic(X) (p11.22)/45,X mosaicism in 1 case, +mar mosaicism in 1 case, 46, XY, del(4) (p15.3) in 1 case, triploidy in 1 case, 45, X in 1 case, and normal karyotypes in 14 cases (Table 6).

**TABLE 6:** Pregnancy outcomes in pregnant women with SUA.

	*N*	Normal	TOP/preterm/death	*χ* ^2^, *p*
iSUA	28	23	5 (4/1/0)	
SUA with ultrasound soft markers	35	26	9 (5/1/3)	0.556, 0.456
SUA with one system malformation	39	28	11 (11/0/0)	0.960, 0.327
SUA with multiple system malformations	29	0	29 (29/0/0)	—

*Note:* Chi‐squared test. A *p*‐value of < 0.05 was considered statistically significant.

Abbreviation: TOP, termination of pregnancy.

## 4. Discussion

With the advancement of modern ultrasonography, prenatal diagnosis of fetal abnormalities has become increasingly common. Notably, SUA is a prenatal ultrasound soft marker associated with elevated risk for chromosomal aneuploidies and fetal malformations. Subsequently, this has raised concerns among obstetricians about whether the occurrence of SUA should be an indication for prenatal chromosomal testing. There is a lack of consensus among these experts, with some arguing that chromosomal testing is unnecessary for pregnant women with SUA, while others claim that SUA is a risk factor for chromosomal abnormalities. In this study, we find that chromosomal evaluation is a necessary component of a prenatal diagnostic plan, given the potential impact of fetal anomalies [8]. Conventional invasive diagnostic procedures, such as amniocentesis and cordocentesis, are considered the gold standard in prenatal diagnosis due to their high accuracy. However, they carry various risks, including bleeding, miscarriage, and potential fetal injury. These risks often impose significant psychological and economic burdens on both the pregnant women and their families [9]. Consequently, there is an urgent need to develop accurate, safe, and effective techniques for conducting prenatal screening for SUA.

Over the past few decades, research has indicated that maternal factors such as race, advanced age, and smoking status represent significant risk factors for SUA [10, 11]. However, these observations are constrained by the availability of large sample sizes, the existence of regional differences, and variations among study populations. Additionally, discrepancies have been reported on the impact of gender on SUA incidence. One study reported no significant gender‐based difference in SUA prevalence [2], while another reported its prevalence among females [11]. In this study, we found that nulliparity (including primigravida and those with a history of miscarriage only) was associated with a higher prevalence of SUA. SUA was more frequently diagnosed before 24 weeks of gestation, reflecting the timing of routine prenatal ultrasound screening rather than a true biological association. Additionally, the incidence of SUA was significantly higher in female fetuses than in male fetuses. The decrease in SUA diagnosis rate after the 24th gestational week may be attributed to detailed ultrasound examinations conducted during the first or second trimester, enabling early detection and management, which subsequently reduces detection rate in the third trimester. Additionally, amniocentesis is typically recommended before 24 weeks, as amniotic fluid culture failure rate increases beyond this gestational age. Therefore, late amniocentesis, at or after 24 weeks of gestation, is primarily used for the prenatal diagnosis of structural abnormalities, especially for cases involving multiple congenital anomalies, with a high diagnostic rate [12]. It should be noted, however, that all 375 cases in this study represented referrals for prenatal diagnosis due to SUA, rather than the total number of pregnant women undergoing prenatal ultrasound, which may introduce potential bias in assessing the relationship between SUA and gestational age. Subsequent studies will incorporate samples of pregnant women undergoing prenatal ultrasound examinations to address this limitation.

Previous studies have reported a strong association between SUA and fetal structural malformations, with approximately 10.9%–28.3% of SUA cases being associated with at least one malformation [4, 13]. In this study, 158 fetuses exhibited related structural malformations, with the most affected systems involving the cardiovascular, genitourinary, and digestive systems, an observation consistent with previous findings [3]. Additionally, the risk of chromosomal abnormalities in fetuses with SUA with structural malformations was found to be higher compared to those in iSUA or SUA with ultrasound soft markers.

Previous studies have reported that the chromosomal abnormality rate for fetuses with any ultrasound soft marker is approximately 4.3%, while the rate specifically for SUA as a single soft marker is 3.17% [14]. Furthermore, the rate for iSUA ranges from 0% to 2.56% [6, 15]. Notably, the chromosomal abnormality rate escalates with increasing concurrent anomalies. Fetuses and neonates with SUA exhibited a 15.35 times greater risk of chromosomal abnormalities, a 6.77 times greater risk of congenital anomalies, and a further 18.7 times greater risk of two or more major malformations [10]. The significant variations observed are attributable to differences in sample size, geographic regions, and other factors. In this study, the karyotype abnormality rate was 16.53%, corroborating the findings in previous studies that indicate that the incidence of chromosomal abnormalities increases with the number of associated malformations [2, 6]. Notably, the chromosomal abnormality rate in the group with multiple system malformations was 37.5%, which was significantly higher compared to that in the other groups. Additionally, the group with a single system malformation exhibited an abnormality rate of 17.02%, which is significantly higher compared to that in the iSUA group. Consequently, chromosomal testing is necessary for fetuses exhibiting SUA with associated malformations, whether multiple or single.

Furthermore, there has been no consensus on whether invasive prenatal diagnosis should be performed in cases of iSUA. Some studies suggest that the likelihood of chromosomal abnormalities in newborns with iSUA is extremely low, and therefore, precludes the need for invasive prenatal diagnosis [16]. It is reported that ultrasound diagnosis of SUA with associated anomalies increases the risk of chromosomal abnormalities, whereas iSUA does not elevate this risk [6]. iSUA is at an increased risk for small for gestational age and pregnancy‐induced hypertension but not for spontaneous preterm birth [17]. A follow‐up study shows no significant differences in longitudinal physical growth or neurologic development between infants with iSUA and normal controls [18]. However, a population‐based study links iSUA to adverse perinatal outcomes, including elevated risks of placental and cord as well as the third stage of labor complications [19]. Although the overall prognosis for iSUA is generally favorable, careful monitoring is warranted due to the potential for coexisting abnormalities. Some congenital malformations may not be detectable until the third trimester or become apparent only after birth [20]. Consequently, heightened clinical vigilance is imperative when managing pregnancies with SUA, factors such as maternal age and reproductive history should be carefully monitored, and a thorough examination of fetal structures should be conducted. For iSUA, noninvasive prenatal screening modalities represent a viable alternative, including serological screening or noninvasive prenatal testing. Conversely, when SUA coexists with structural abnormalities, invasive prenatal diagnostics should be recommended, such as karyotyping and SNP‐array testing, mandating immediate initiation following ultrasound diagnosis. Hence, first‐ and second‐trimester ultrasound screening assumes critical importance in this diagnostic cascade.

Although the risk of chromosomal abnormalities in iSUA is low, the risk of trisomy 18 remains 3–5 times higher compared to the general population [21]. In this study, chromosomal abnormalities were observed in both the iSUA group and the group with ultrasound abnormalities, with trisomy 18 being the most common.

Our study has several limitations. SNP‐array was performed on only 152 fetuses due to technical constraints before June 2016, limiting subgroup comparisons. The follow‐up rate was also limited by loss of contact in some cases. Despite these limitations, our study provides two clinically actionable findings. First, SNP‐array detected 13 microdeletions/microduplications (9 pathogenic, representing a 5.9% incremental yield over karyotyping), including in single‐system malformations, supporting broader use of chromosomal microarray analysis (CMA). Second, 3 of 101 iSUA cases (2.97%) had chromosomal abnormalities (2 trisomy 18, 1 inv (9)), all terminated, challenging the assumption that iSUA is entirely benign. Larger prospective studies are needed to validate these findings.

While SNP‐array technology exhibits comparable detection efficiency for aneuploidy and unbalanced karyotype rearrangements to traditional karyotyping methods, it offers higher resolution and sensitivity. Additionally, SNP is an effective genetic diagnostic tool, especially in cases where prenatal ultrasound identifies structural abnormalities in the fetus, since it can detect a range of clinically significant genomic CNVs [21–23]. Given that SNP‐array testing does not require cell culturing, it is particularly useful for samples where culturing is challenging, or for samples with significant symptoms but no abnormalities detected through conventional karyotyping. Chromosomal microarray technology in fetuses with ultrasound abnormalities has garnered significant application, thereby substantially improving the detection of chromosomal microdeletion or microduplication syndromes [24, 25].

While SNP‐array technology shows significant advantages in detecting pathogenic CNVs, challenges remain limitations. It is vital in detecting chromosomally balanced translocations and inversions, but it offers limited capability in interpreting the pathogenicity of numerous small chromosomal segments. Given that SUA is a significant indicator in prenatal screening, both conventional karyotyping and SNP‐array testing should be used to analyze it to provide more comprehensive genetic information. This dual approach exhibits the potential to enhance both clinical decision‐making for SUA prognosis assessment and genetic counseling.

This study has additional limitations inherent to its retrospective design. First, the sample size was limited, which may introduce selection bias and potentially influence the results. Second, some of the case data was incomplete, with some failing to undergo SNP‐array testing due to technical limitations. Additionally, the follow‐up period was limited by loss of contact or lack of cooperation from patients, which may introduce bias in assessing neonatal outcomes. Nonetheless, this study exhibits notable advantages involving its assessment of various SUA categories, including iSUA, SUA with ultrasound soft markers, and SUA with single or multiple system malformations. Additionally, we explored the association between SUA, fetal malformations, and chromosomal abnormalities, which may provide valuable insights for prenatal diagnosis.

## 5. Conclusions

This study demonstrated that SUA is most prevalent among pregnant women aged 25–35, and the incidence was significantly higher in nulliparous women (including primigravidas and those with a history of miscarriage only). Additionally, SUA is more common in female fetuses. Furthermore, when an SUA is identified on prenatal ultrasound—particularly in conjunction with other structural or soft marker abnormalities—an invasive prenatal diagnostic procedure should be recommended to evaluate potential chromosomal or genetic anomalies. Notably, SUA is a potential marker for fetal developmental abnormalities and may be influenced by a combination of technical, genetic, and environmental factors. Its pathogenesis may involve abnormal embryonic vascular development and epigenetic regulation. Additionally, SUA is significantly associated with cardiovascular and genitourinary malformations, particularly when chromosomal abnormalities are present, leading to a poorer prognosis. Future research utilizing advanced molecular diagnostic techniques may help elucidate the underlying molecular mechanisms of SUA and support the development of individualized, risk‐based prenatal management strategies.

## Author Contributions

Yuqin Chen and Xiaoqing Wu conceived and designed the research, wrote the original draft, and prepared tables. Meiying Cai, Linjuan Su, and Xiaorui Xie conducted data gathering and management. Hailong Huang, Rongzhao Zhang, and Liangpu Xu were responsible for commenting and editing.

## Funding

This study was supported by the Startup Fund for Scientific Research of Fujian Medical University (2022QH1193).

## Ethics Statement

This study was approved by the Protection of Human Ethics Committee of Fujian Provincial Maternity and Children’s Hospital (2014–042). All procedures were conducted under the principles of the Declaration of Helsinki. Written informed consent was obtained from all individual participants before being enrolled in the study.

## Consent

The authors have nothing to report.

## Conflicts of Interest

The authors declare no conflicts of interest.

## Data Availability

The datasets used and/or analyzed during the current study are available from the corresponding author on reasonable request.
